# An fMRI study examining the role of the extended amygdala and amygdala in emotion and inhibitory control in native versus second language processing

**DOI:** 10.1371/journal.pone.0310129

**Published:** 2024-11-04

**Authors:** Maya Sugita-McEown, Michiru Makuuchi, Taiga Naoe, James Ellinger, W. L. Quint Oga-Baldwin, Kristopher McEown

**Affiliations:** 1 Faculty of Education and Integrated Arts and Sciences, Waseda University, Tokyo, Japan; 2 Research Institute of the National Rehabilitation Center for Persons with Disabilities, Tokorozawa City, Saitama Japan; 3 Department of Foreign Languages, Nippon Medical School, Tokyo, Japan; 4 Faculty of Science and Engineering, Waseda University, Tokyo, Japan; Juntendo University, JAPAN

## Abstract

The role of the extended amygdala and amygdala in mediating emotion and inhibitory control in native language versus second language processing is currently not well understood. The current study examined activity in the extended amygdala and amygdala when twelve healthy young adults were exposed to emotional-linguistic stimuli in either their native language (i.e., Japanese) or in a second language (i.e., English) using a go/no-go task while undergoing fMRI scans. Data was bootstrapped using random resampling. A significant interaction was observed for the amygdala and extended amygdala activity for language (English vs. Japanese), emotional-linguistic valence (positive, negative, neutral) and inhibitory control (go/no-go condition). Furthermore, main effects were observed for language and valence for the amygdala and extended amygdala. Main effects were observed for inhibitory control for the extended amygdala and right amygdala but not for the left amygdala, which did not show a main effect for inhibitory control. Significant interactions and main effects were also observed for behavioral outcomes (go/no-go reaction time and accuracy scores) for the amygdala and extended amygdala. Post hoc analyses found that under conditions of inhibitory control participants had less activation in the extended amygdala and amygdala when processing emotional information in English (i.e., second language) compared to Japanese (i.e., native language). In summary, our findings suggest that the amygdala and extended amygdala may mediate emotion and inhibitory control when participants process information in their native (Japanese) versus a second language (English).

## Introduction

The extended amygdala is a neural continuum located in the forebrain that includes the central nucleus of the amygdala and the bed nucleus of the stria terminalis as major nodes [1, 2]. The amygdala consists of thirteen nuclei in total, including the medial, central, basolateral, basomedial and lateral nuclei [3]. The extended amygdala and amygdala mediate similar and distinct processes. For example, both the extended amygdala and amygdala mediate fear responses via the central nucleus of the amygdala [4]. While the extended amygdala may play an important, separate, role in processing changes to environmental cues, the amygdala may play a distinct role in mediating the coding of emotions from sensory information [5]. Past studies have separately examined the role of the extended amygdala and amygdala in mediating emotion [2, 6]. For example, research suggests that the processing of positive and negative emotional stimuli is mediated by the extended amygdala [7] and the amygdala [8, 9]. Bonnet and colleagues [8] showed photos presenting emotionally positive stimuli and observed higher activation in the right and left amygdala that was positively associated with the emotional intensity of the presented stimuli. In addition, a meta-analysis by Sabatinelli and colleagues [10] of 157 fMRI studies found that increased right and left amygdala activation was associated with presentations of emotionally positive facial expressions or stimuli depicting pleasant scenes. Furthermore, Costa and colleagues [11] had participants imagine emotional (pleasant or unpleasant) scenes while undergoing fMRI scans. They found that increased amygdala activity occurred when participants imagined both pleasant and unpleasant scenes and there were non-significant differences in amygdala activity between the two emotional valances (i.e., pleasant versus unpleasant scenes). For the extended amygdala, Liberzon and colleagues [7] presented participants with pleasant, unpleasant, or neutral images while obtaining PET scans. They found that the extended amygdala activity was significantly increased when participants viewed pleasant or unpleasant visual stimuli.

Research suggests that the right and left amygdala may mediate differently negative and positive emotions. Specifically, Lanteaume and colleagues [12] electrically stimulated the right and left amygdala in human patients and found that electrical stimulation of the right amygdala elicited negative emotions (e.g., sadness, fear); whereas electrical stimulation of the left amygdala elicited both positive emotions (e.g., happiness, joy) and negative emotions (e.g., anxiety, fear, sadness). Moreover, Bujarski and colleagues [13] found that disruptive electrical stimulation of the left amygdala was associated with a reduction in valence ratings of emotionally positive and negative visual stimuli. Specifically, emotionally positive, and emotionally negative visual stimuli were rated as more neutral after electrical stimulation to the left amygdala whereas the valence ratings of neutral visual stimuli were not affected by electrical stimulation. Hamann and colleagues [14] used PET scans to examine left or right amygdala activation in response to positive or negative visual stimuli (i.e., photographs). They observed increased left amygdala activation when participants were exposed to positive and negative visual stimuli; however, increased right amygdala activation was observed only in response to negative visual stimuli. In summary, these data suggest that the right amygdala may mediate negative emotion, while the left amygdala may mediate both positive and negative emotion.

### Inhibitory control, amygdala/extended amygdala function and emotional-linguistic processing

Inhibitory control, a type of executive function, is associated with the ability to regulate emotion [15, 16] and go/no-go tasks are used extensively to measure inhibitory control via response inhibition [17]. Moreover, go/no-go tasks have been used to examine the ability of individuals to regulate their responses to several types of emotional stimuli including faces [18] or words [19]. Some evidence suggests that the amygdala and extended amygdala may play a role in mediating inhibitory control. A study by Depue and colleagues [20] examined patients with post-traumatic stress disorder and found that impaired inhibitory control (measured using a go/no-go task) was associated with volume reduction in the left amygdala. In addition, Silbersweig and colleagues [21] used an emotional-linguistic go/no-go task in which borderline personality disorder patients were shown positive, negative, and neutral words presented in only their native language (i.e., English). They found increased bilateral extended amygdala activation associated with exposure to negative linguistic stimuli during an inhibitory control condition (i.e., the negative word no-go condition).

However, no studies have examined the possible interaction of language (i.e., native versus second), amygdala/extended amygdala function and emotional self-regulation (i.e., inhibitory control). This is important to investigate because if participants display less amygdala and/or extended amygdala activation and display better emotional self-regulation (i.e., better performance in an inhibitory control task) in a second language compared to a native language, this may provide evidence for an advantage to using a second language for tasks that, for example, require self-regulation in emotionally taxing situations compared to completing the same task in a native language.

Our study has two primary aims. The first aim of our research is to examine to what effect positive or negative emotional-linguistic stimuli might have on amygdala and/or extended amygdala activation when the linguistic stimuli is presented in the participant’s native language (i.e., Japanese), or a second language (i.e., English). Some evidence suggests that participants may have differing amygdala activation when exposed to emotional stimuli in their native language compared to a second language. Nakic and colleagues [22] found, using fMRI, that when negative emotional words were presented in the participant’s native language, they had greater bilateral amygdala activation compared to presentations of emotionally neutral words. Furthermore, measures of skin conductance responses (SCR), which according to Laine and colleagues [23] are mediated by the amygdala, found higher SCRs in response to presentations of emotionally negative words compared to emotionally neutral words in native English speakers whereas SCR increases to emotionally negative English words were not observed in non-native English speakers [24]. Hsu, Jacobs and Conrad [25] had participants read passages meant to elicit positive, neutral, or negative emotions in either their native language (i.e., German) or in a second language (i.e., English). They found greater bilateral amygdala activation to emotionally positive text compared to emotionally neutral text, but only in their native language.

The second aim of our research is to examine differences in inhibitory control between Japanese and English emotional-linguistic stimuli. Specifically, we investigate whether differences in the ability to engage in inhibitory control (a correlate of emotional regulation) may occur when participants are presented with emotional-linguistic stimuli presented in their native language (i.e., Japanese) or in a second language (i.e., English) by using an emotional-linguistic go/no-go task.

### Present study

In the current study, we examine the role of the extended amygdala and amygdala in emotion and inhibitory control using an emotional-linguistic go/no-go task that exposed participants to emotionally valenced stimuli in their native language (i.e., Japanese) or in a second language (i.e., English) while undergoing fMRI scans. We will also examine the extent to which inhibitory control may be affected when participants are exposed to emotional-linguistic stimuli in their native language or in a second language. That is, go/no-go accuracy scores (i.e., errors of commission) and reaction time will be compared between Japanese and English emotional-linguistic stimuli conditions.

## Materials and methods

### Participants

Data were collected from 12 healthy young adults (8 females; mean age = 20.3 years; range = 19–21 years) who were attending a private university in Tokyo and learning English as a second language. All participants were native Japanese speakers.

TOEFL ITP scores were obtained from ten participants with a mean of 530.8 (SD = 21.3). All participants reported the number of years that they had spent learning English with a mean of 10.8 years (SD = 2.82) and the number of minutes per week spent studying English (mean = 182.5; SD = 116.1). Four participants reported studying abroad in an English-speaking country for a mean of 55.2 days (SD = 48.5). Research ethics approval was obtained from the “University Ethics Review Committee on Research with Human Subjects” and the research ethics committee of the National Rehabilitation Center for Persons with Disabilities in Japan. Written consent was obtained from all participants prior to data collection. The recruitment period for the study began December 13, 2019, and ended January 28, 2020. No participants indicated a history of neuropsychiatric disorders, and all had normal or corrected-to-normal vision.

### Emotional-linguistic go/no-go task

**Fig 1:**
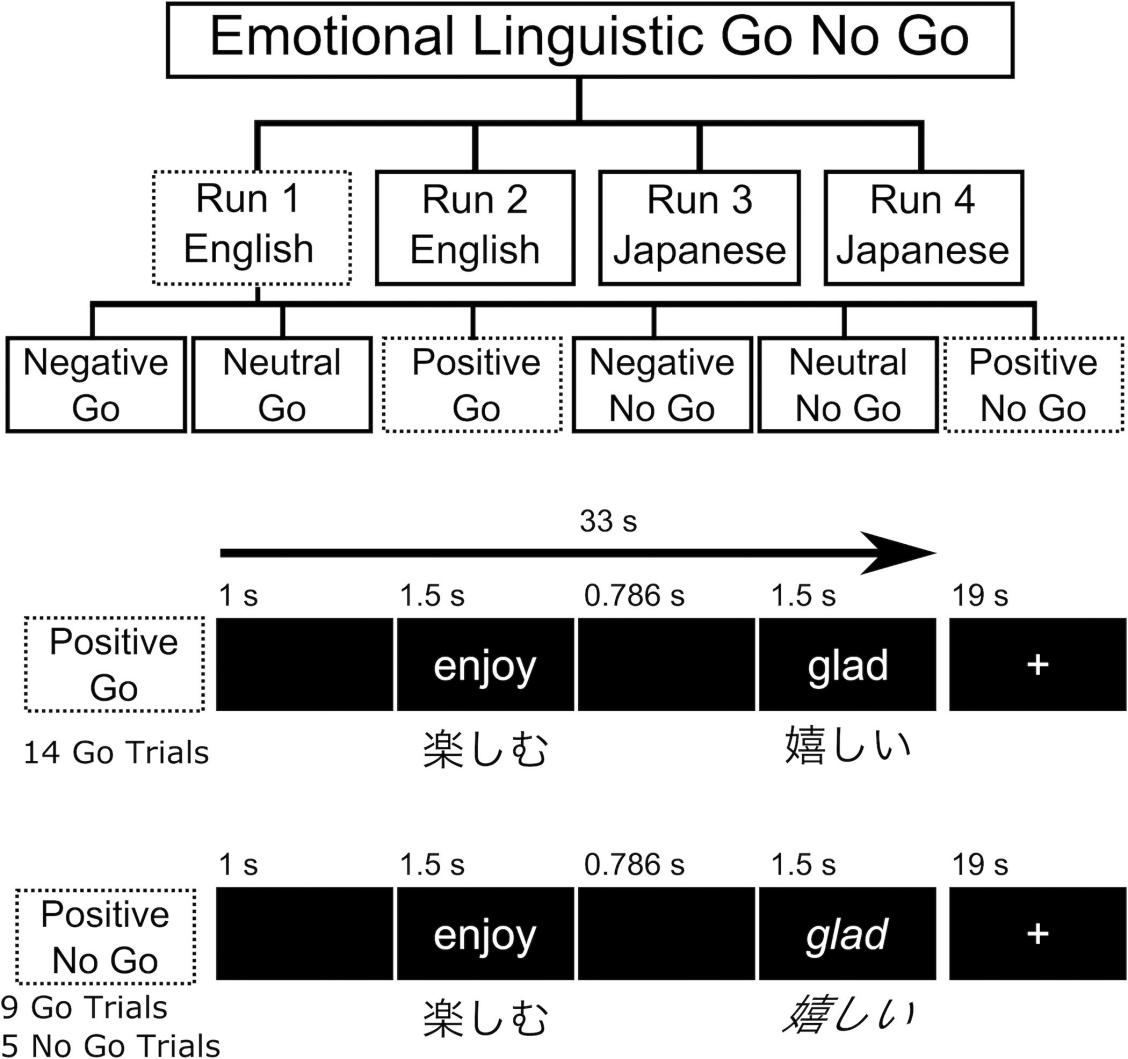
Flow diagram illustrating the emotional linguistic go/no-go procedure. Italics indicate no-go trials. Dotted lines highlight an example Run (i.e., English Run 1) and trial blocks (e.g., positive go) used to illustrate trial times for each stimulus presentation.

The emotional-linguistic go/no-go task used in the present study was adapted from Goldstein and colleagues [19]. A flow diagram illustrating the modified emotional-linguistic go/no-go procedure is illustrated in Fig 1. The emotional-linguistic go/no-go task was presented to participants using Presentation software from Neurobehavioral Systems (https://www.neurobs.com/, Berkeley, CA). The task was presented with 6 blocks per run, using 14 words per block and 4 runs in total (Fig 1). For half of the participants, the first two runs were presented in English and the second two runs were presented in Japanese. For the other half of the participants, this order was reversed. An example of one English run is shown and the matched Japanese words that were presented in a Japanese run are also displayed in Fig 1.

Instructions were provided in Japanese, and participants were instructed to press a button as quickly as possible if a word appeared in normal style, which represents a go trial, and to refrain from pressing the button if the words appeared in italic style, which represents a no-go trial. Each word was presented as black text on a light grey background for 1.5 seconds followed by an interstimulus interval of 0.7 seconds. The total duration of a block was 33 seconds followed by a 19 second break during which a fixation cross appeared. English and Japanese words used in the emotional-linguistic go/no-go task were balanced between the positive, neutral, and negative emotional word conditions for imageability, word length, frequency, and part of speech. Words in each block (e.g., positive English words presented in the positive go block, see Fig 1) were presented in a randomized order as per Goldstein and colleagues [19]. After finishing the go/no-go task participants completed a word comprehension test, outside the scanner room, containing all the English words presented in the task to ensure that participants understood the English words used in the task. Specifically, participants were asked to circle any English words from the list for which they did not understand the meaning. All participants indicated that they understood the meaning of the English words based on the results from the word comprehension test (see Table 1 for a list of English and Japanese words used in the experiment).

**Table 1:** English and Japanese words used in the experiment.

Positive		Neutral		Negative	
beautiful	美しい	year	年	afraid	恐れて
celebrate	祝う	remember	思い出す	angry	怒って
enjoy	楽しむ	local	地方の	danger	危険
excited	興奮して	example	例	death	死
friend	友達	write	書く	evil	邪悪な
friendly	友好的な	understand	理解する	fear	恐怖
fun	楽しい	size	大きさ	hate	ひどく嫌う
glad	嬉しい	today	今日	kill	殺す
good	良い	city	都市	pain	痛み
happy	幸せな	language	言語	sad	悲しい
hug	抱きしめる	air	空気	sick	病気
joy	喜び	age	年齢	violent	暴力的な
kind	親切な	tall	背が高い	war	戦争
love	愛する	begin	始める	worry	心配
pleasure	楽しみ	connect	接続する	defeat	敗北
share	共有する	walk	歩く	defeated	負けの
smile	笑顔	minute	分	destroy	破壊する
success	成功	delete	消す	embarrassed	恥ずかしい
peace	平和	bottle	瓶	fail	失敗する
softly	柔らかく	famous	有名な	lose	失う
music	音楽	tiny	小さい	loss	損失
proud	誇りに思って	sentence	文	nervous	緊張して
cheerful	元気のいい	locate	位置する	rejected	却下された
calm	落ち着いた	tobacco	タバコ	ugly	醜い
accomplishment	達成	organize	組織する	bored	退屈して
pretty	可愛い	noon	昼	lonely	孤独な
thankful	感謝して	educate	教育する	worried	心配して
polite	丁寧な	wipe	拭く	annoyed	いらいらして

### fMRI data acquisition

fMRI scans were used to measure neurophysiological responses to positive, neutral, and negative emotional-linguistic stimuli presented in participants’ native language (i.e., Japanese) or in a second language (i.e., English) using the modified version of the emotional-linguistic go/no-go task described above. The MRI data were collected with a 3T MRI scanner (MAGNETOM Skyra; Siemens, Erlangen, Germany). T1-weighted high-resolution structural images were acquired (MPRAGE sequence, TR = 1000 ms, TE = 2.98, inversion time = 900 ms, flip angle = 9°, field of view = 256×256 mm, matrix 256×256, sagittal 224 slices, 1mm isotropic resolution). Three hundred twenty-one functional scans were obtained for each experimental session with a multiband-echo-planner imaging (EPI) sequence [26–28] (multiband factor = 2, repetition time = 1000 ms, echo time = 30 ms, flip angle = 68, field of view = 192×192 mm, matrix 64×64, 30 axial slices, slice thickness = 3mm with 1mm gap). The slices were aligned to the AC-PC plane, covering the entire brain.

### Preprocessing of fMRI data

We used the SPM12 software package (available at https://www.fil.ion.ucl.ac.uk/spm/) for preprocessing of structural and functional MRI data. The functional images were realigned to the mean image, and the difference of slice acquisition timing was corrected. Then, the functional images were co-registered to individuals’ anatomical images. The spatial normalization to the East Asian brain template in SPM12 was processed in two steps: 1) estimating the normalization parameters and 2) writing the normalized images with the parameters. All functional images were transformed into the Montreal Neurological Institute (MNI) stereotaxic space to allow for multi-subject analyses. The functional images were resampled into 3×3×3mm^3^ voxels with the seventh degree B-spline interpolation and smoothed with a 6-mm full width at half maximum (FWHM) Gaussian kernel.

### fMRI data analysis

Since we had specific interests in the role of the amygdala and extended amygdala, we performed region of interest (ROI) analyses using SPM12 (https://www.fil.ion.ucl.ac.uk/spm/) and Marsbar 0.44 (https://marsbar-toolbox.github.io/). The condition effects in each ROI were estimated with a block design per participant by a general linear model. The regressors for the twelve target conditions (i.e., Language (English/Japanese) x Task (go/no-go) x Valance (neutral/positive/negative) were modelled as box-car functions which started and ended with experimental blocks of a duration of 33 s in the design matrix. The six motion parameters were also included in the design matrix as covariates of no interest to account for motion-related variance. Low frequency noise was removed using a high-pass filter with a cut-off period of 128 s. We estimated temporal correlations in fMRI time series using an autoregressive AR(1) model in estimation of parameters, which were used to correct for non-sphericity during statistical inference.

We performed analyses on the following four ROIs defined as 6 mm radius spheres centered at the following coordinates: the right extended amygdala (REA; 21, 6, -12), left extended amygdala (LEA; -18, 6, -9), the right amygdala (RA; 18, -6, -18), and the left amygdala (LA; -24, -7, -17). These coordinates were obtained from the following sources: Silbersweig and colleagues [21] for the REA and LEA, Perez and colleagues [29] for the RA, and Kark and colleagues [30] for the LA. The mean beta value in these ROIs was calculated for individual participants.

### Statistical analyses

Given the small size of this sample, data was bootstrapped using randomized resampling to create a resampled population of 10000 using Stata version 18 software (StataCorp. 2023. Stata Statistical Software: Release 18. StataCorp LLC.). Bootstrapping has been indicated as a powerful solution for generalizing small samples in fMRI studies [31] and offers some safeguards against errors and random noise in the data.

After completing the bootstrap procedures, a within-subjects analysis of variance (ANOVA) was conducted to examine the effects of three categorical factors: English vs. Japanese, go vs. no-go condition, emotional-linguistic valence (positive, negative, neutral) on participants accuracy of response, reaction times, and activity in the left and right amygdalae, and activity in both extended amygdalae. Finally, Tukey’s HSD post hoc tests were used to examine whether differences in the ability to engage in inhibitory control (i.e., during the no-go condition) might occur when participants are presented with emotional-linguistic stimuli presented in Japanese or English. That is, accuracy scores, reaction time and differences in activity for each ROI were examined in the no-go or go conditions between languages for each emotional valence (i.e., negative, neutral, positive) with a threshold for significance established at p < .001.

## Results

### MRI data analyses

Data from two participants were excluded from the final analysis because their BOLD responses were more than three standard deviations from the mean in the Japanese and English negative word conditions. Therefore, ten participants remained for the final data analysis. Participant resampling rates are presented in Table 2.

**Table 2:** Bootstrapped resample frequencies for each participant.

Participant Number	Frequency
1	1042
2	1068
3	1080
4	876
5	842
6	1356
7	1080
8	1090
9	788
10	778

**Fig 2:**
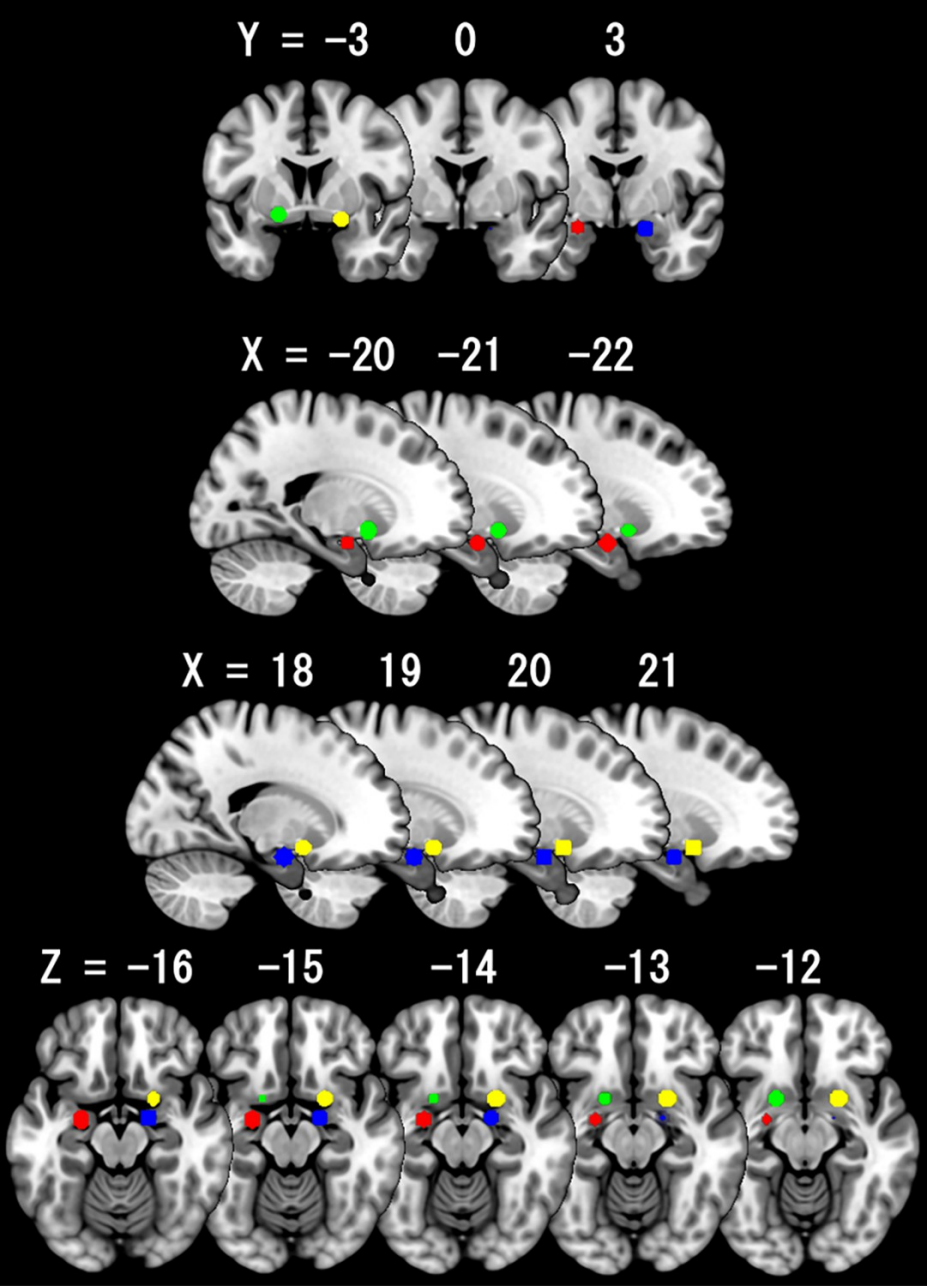
Anatomical topography for the left amygdala (green), right amygdala (yellow), left extended amygdala (red), and right extended amygdala (blue).

Please see Fig 2 for anatomical topography of each ROI and S1 Fig for EPI signals for each ROI.

### Emotional word exposure and amygdala activity

**Fig 3:**
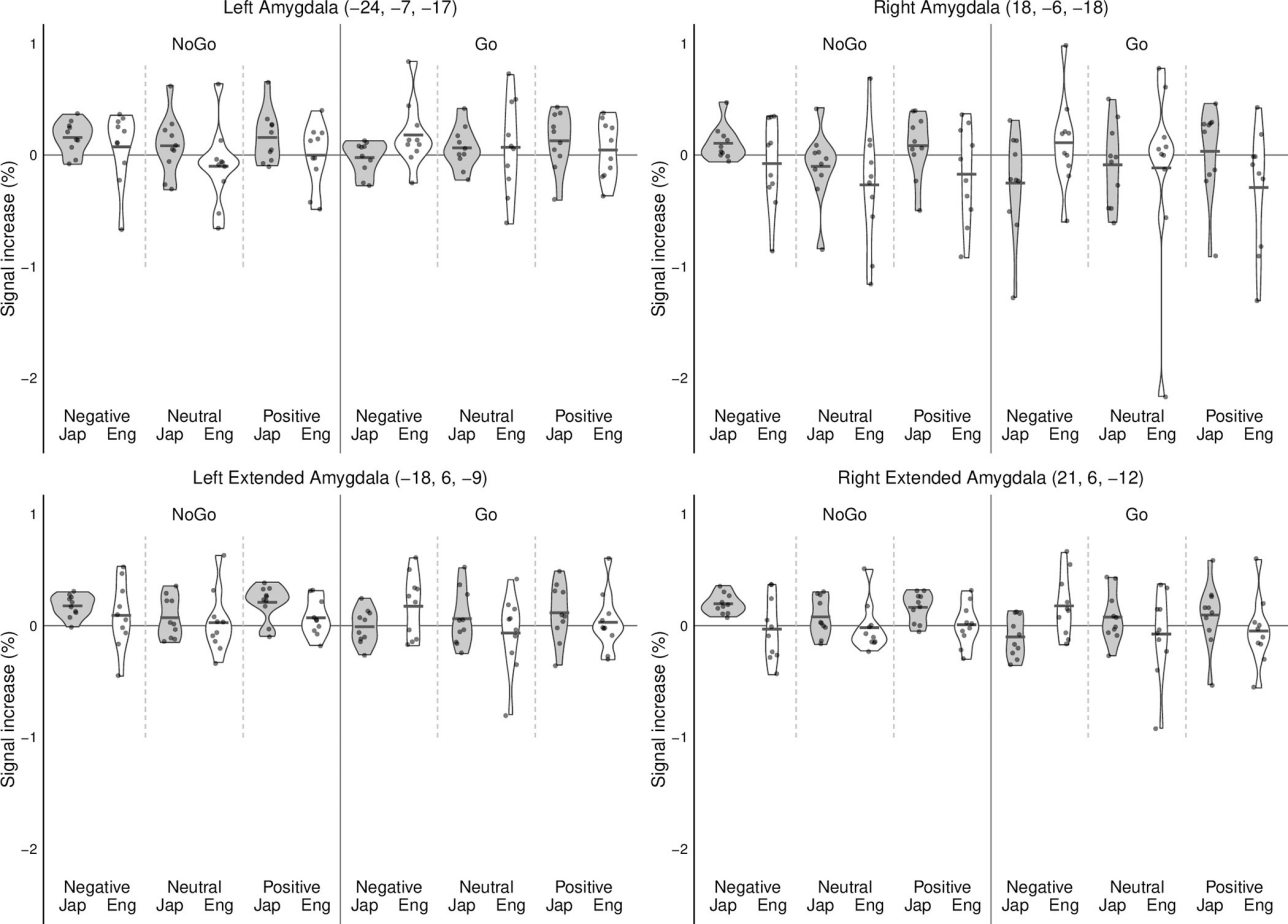
BOLD activity in the left amygdala, right amygdala, left extended amygdala and right extended amygdala for language (English/Japanese), word valence (negative/neutral/positive) and go/no-go condition. Each dot represents one participant. The thick black horizontal bar represents the average signal increase for each condition.

For activation in the left amygdala, the overall model was significant, F(20, 5053) = 139.99, p > .001, R^2^ = .356 (see Fig 3).

Results revealed a significant main effect of language condition, F(1, 5063) = 211.80, p < .001, η^2^ = .0328, indicating differential activation in the left amygdala between English and Japanese instructions. However, no significant main effect was found for the go/no-go condition, F(1, 5063) = 0.17, p = .6822, suggesting that left amygdala activation did not vary significantly between conditions where participants were instructed to respond or withhold response. Word valence demonstrated a significant main effect, F(2, 5062) = 33.30, p < .001, with a small effect size, η^2^ = .0106, revealing differences in left amygdala activation across negative, neutral, and positive word stimuli. Interaction effects were detected for each of the conditions, again with minute effects: word valence x go/no-go condition x language, F(2, 5053) = 74.13, p < .001, η^2^ = .02; word valence x language, F(2, 5053) = 170.91, p < .001, η^2^ = .04; go/no-go x language, F(2, 5053) = 209.39, p > .001, η^2^ = .03, go/no-go x word valence, F(2, 5053) = 127.93, p < .001, η^2^ = .03. Tukey’s HSD post hoc comparisons of emotional valence between each language in the no-go condition showed significantly increased left amygdala activity for Japanese words compared to English words for negative (Japanese Mean = 0.205; English Mean = 0.112; p < .001), neutral (Japanese Mean = 0.127; English Mean = -0.194; p < .001), and positive (Japanese Mean = 0.037; English Mean = -0.061; p < .001) emotion conditions (see Fig 3). However, in the go condition a significant decrease in left amygdala activity was observed for Japanese words compared to English words in the negative emotion condition (Japanese Mean = -0.015; English Mean = 0.095; p < .001). Whereas an increase in left amygdala activity was observed in Japanese compared to English neutral (Japanese Mean = 0.128; English Mean = 0.080; p < .001) and positive (Japanese Mean = 0.139; English Mean = -0.031; p < .001) words (see Fig 3).

Right amygdala activation held similar results (see Fig 3). The overall model was also significant, F(20, 5053) = 139.99, p > .001, R^2^ = .382. Results revealed a significant main effect of language condition, F(1, 5063) = 269.56, p < .001, with a moderate effect size, η^2^ = .0388, indicating differences in activation levels between English and Japanese instruction sets. In addition, the go/no-go condition yielded a significant main effect, F(1, 5063) = 33.51, p < .001, η2 = .02. Word valence also demonstrated a significant main effect, F(2, 5062) = 57.94, p < .001, with a small effect size, η^2^ = .0172, revealing differences in right amygdala activation across negative, neutral, and positive word stimuli. Interaction effects were detected for each of the conditions, again with minute effects: word valence x go/no-go condition x language, F(2, 5053) = 119.48, p < .001, η^2^ = .03; word valence x language, F(2, 5053) = 155.07, p < .001, η^2^ = .04; go/no-go x language, F(2, 5053) = 71.39, p > .001, η^2^ = .01, go/no-go x word valence, F(2, 5053) = 36.39, p < .001, η^2^ = .01. Similar to the left amygdala, post hoc comparisons of emotional valence between each language in the no-go condition showed significantly increased right amygdala activity for Japanese words compared to English words for negative (Japanese Mean = 0.078; English Mean = -0.114; p < .001), neutral (Japanese Mean = 0.009; English Mean = -0.460; p < .001) and positive (Japanese Mean = -0.042; English Mean = -0.200; p < .001) emotion conditions (see Fig 3). Moreover, a similar pattern of activity was observed for the right amygdala in the go condition, that is a significant decrease in right amygdala activity was observed for Japanese words compared to English words in the negative emotion condition (Japanese Mean = -0.176; English Mean = 0.017; p < .001). However, an increase in right amygdala activity was observed in Japanese compared to English neutral (Japanese Mean = -0.023; English Mean = -0.202; p < .001) and positive (Japanese Mean = 0.014; English Mean = -0.519; p < .001) words (see Fig 3).

### Emotional word exposure and extended amygdala activity

Left extended amygdala activity showed complementary results, with a significant overall model, F(20, 5053) = 11.52, p > .001, R^2^ = .306 (see Fig 3). Language condition demonstrated a significant main effect, F(1, 5063) = 185.40, p < .001, with a small effect size, η^2^ = .0309, indicating differences in activation between English and Japanese instructions. Similarly, the go/no-go condition exhibited a significant main effect, F(1, 5063) = 148.66, p < .001, with a small effect size, η^2^ = .0250, suggesting variation in activation between response conditions. Word valence displayed a significant main effect, F(2, 5062) = 60.76, p < .001, with a small effect size, η^2^ = .0205, revealing differences in activation across negative, neutral, and positive word stimuli. Minute interaction effects were seen for each of the conditions: word valence x go/no-go condition x language, F(2, 5053) = 50.17, p < .001, η^2^ = .01; word valence x language, F(2, 5053) = 212.94, p < .001, η^2^ = .06; go/no-go x language, F(2, 5053) = 129.29, p > .001, η^2^ = .02, go/no-go x word valence, F(2, 5053) = 44.37, p < .001, η^2^ = .01. For the left extended amygdala, post hoc comparisons of emotional valence between each language in the no-go condition showed a similar pattern of results compared to the left and right amygdala. Specifically, increased activity was observed for Japanese words compared to English words for the negative (Japanese Mean = 0.214; English Mean = 0.133; p < .001), neutral (Japanese Mean = 0.149; English Mean = -0.084; p < .001), and positive (Japanese Mean = 0.176; English Mean = 0.032; p < .001) emotion conditions (see Fig 3). For the go condition a significant decrease in left extended amygdala activity was observed for Japanese words compared to English words in the negative emotion condition (Japanese Mean = -0.014; English Mean = 0.124; p < .001) and, again, an increase in left extended amygdala activity was observed in Japanese compared to English neutral (Japanese Mean = 0.125; English Mean = -0.102; p < .001) and positive (Japanese Mean = 0.094; English Mean = -0.027; p < .001) words (see Fig 3).

Finally, right extended amygdala results were also significant, F(20, 5053) = 11.52, p > .001, R^2^ = .306 (see Fig 3). Results indicated a significant main effect of language condition, F(1, 5063) = 555.67, p < .001, with a medium effect size, η^2^ = .0860, suggesting differing activation levels between English and Japanese instructions. Similarly, the go/no-go condition exhibited a significant main effect, F(1, 5063) = 129.72, p < .001, with a small effect size, η^2^ = .0217, indicating variation in activation between response conditions. Word valence demonstrated a significant main effect, F(2, 5062) = 15.51, p < .001, with a small effect size, η^2^ = .0053, revealing differences in activation across negative, neutral, and positive word stimuli. As with the other results, interaction effects were significant but very small: word valence x go/no-go condition x language, F(2, 5053) = 50.17, p < .001, η^2^ = .05; word valence x language, F(2, 5053) = 212.94, p < .001, η^2^ = .05; go/no-go x language, F(2, 5053) = 129.29, p > .001, η^2^ = .03; go/no-go x word valence, F(2, 5053) = 44.37, p < .001, η^2^ = .01. Similarly, for the right extended amygdala post hoc comparisons of emotional valence between each language in the no-go condition showed increased activity for Japanese words compared to English words for the negative (Japanese Mean = 0.212; English Mean = -0.035; p < .001), neutral (Japanese Mean = 0.141; English Mean = -0.130; p < .001), and positive (Japanese Mean = 0.145; English Mean = -0.053; p < .001) emotion conditions (see Fig 3). For the go condition, a significant decrease in right extended amygdala activity for Japanese compared to English words was observed in the negative emotion condition (Japanese Mean = -0.087; English Mean = 0.080; p < .001) and increased right extended amygdala activity in Japanese compared to English neutral (Japanese Mean = 0.121; English Mean = -0.141; p < .001) and positive (Japanese Mean = 0.049; English Mean = -0.152; p < .001) words (see Fig 3).

### Emotional word exposure and contrasting ROI activity

Contrasting ROI activity (i.e., left amygdala, right amygdala, left extended amygdala, and right extended amygdala) were compared for all variables under consideration to examine potential differences in activity between ROIs. A significant main effect for ROI was observed, F(3, 15186) = 1445.34, p < .001, η^2^ = .22. Interaction effects were also significant but small: ROI x language, F(3, 15186) = 131.20, p < .001, η^2^ = .02; ROI x go/no-go, F(3, 15186) = 90.22, p < .001, η^2^ = .01; ROI x word valence, F(6, 15186) = 38.24, p < .001, η^2^ = .01; ROI x language x go/no-go, F(3, 15186) = 8.18, p < .001, η^2^ = .002; ROI x language x word valence, F(6, 15186) = 36.25, p < .001, η^2^ = .01; ROI x go/no-go x word valence, F(6, 15186) = 62.14, p < .001, η^2^ = .02; ROI x language x go/no-go x word valence, F(6, 15186) = 114.47, p < .001, η^2^ = .04.

### Behavioral results

**Fig 4:**
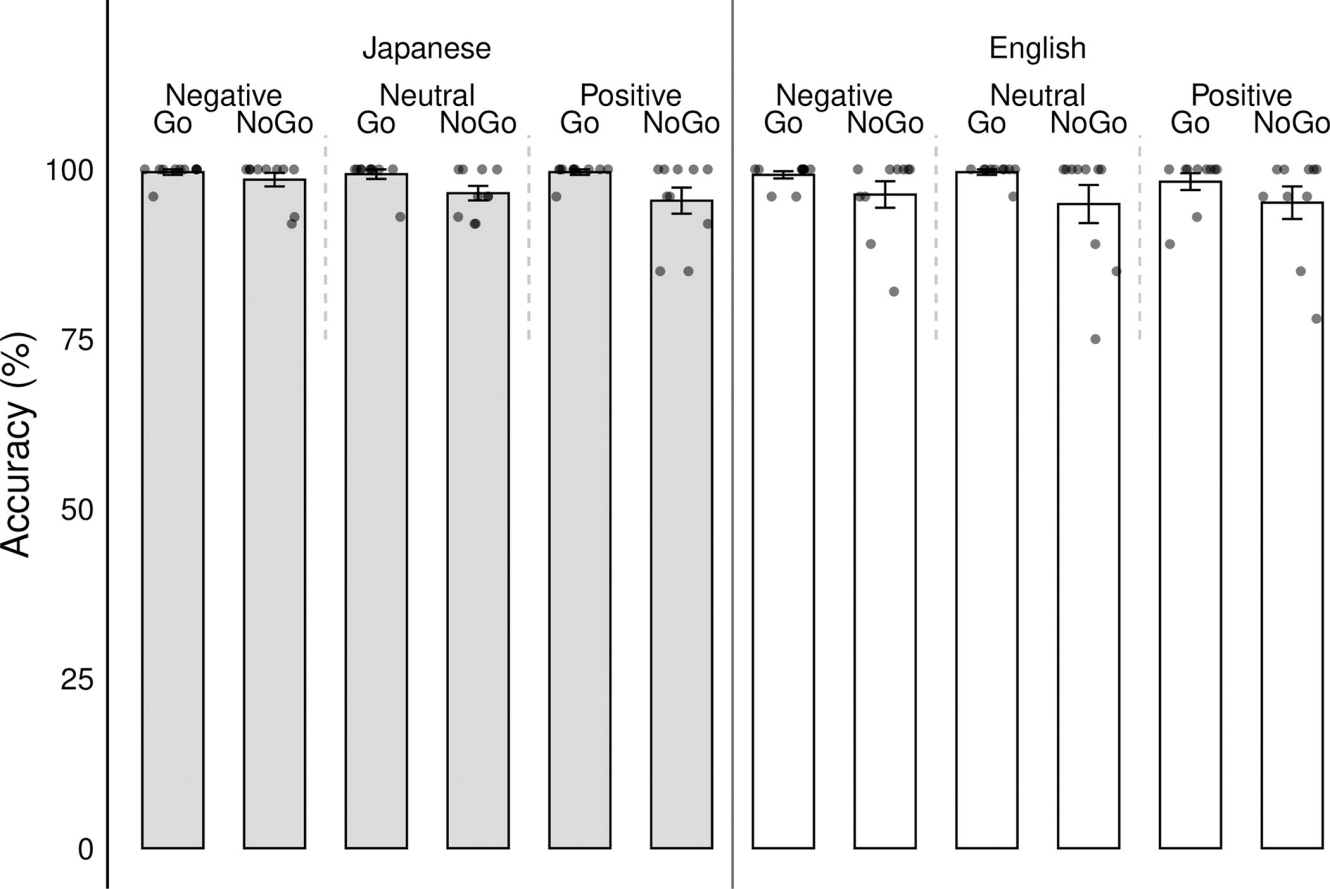
Accuracy (0–100 percent) of each participant for language (English/Japanese), word valence (negative/neutral/positive) and go/no-go condition. Error bars denote standard errors of the mean. Each dot represents one participant.

For behavior (see Fig 4), results of the conditions on score accuracy showed statistically significant differences.

The overall model was significant and explained roughly 44% of the variance (R^2^ = .436). For the language condition, there was a significant main effect, F(1, 9999) = 256.71, p < .001, η^2^ = .025, indicating that participants performed differently depending on whether instructions were presented in English or Japanese. Similarly, for the go/no-go condition, there was a significant main effect, F(1, 9999) = 1247.65, p < .001, η^2^ = .076. Regarding word valence, there was also a significant main effect, F(2, 9988) = 53.31, p < .001, η^2^ = .007, indicating that participants’ accuracy scores differed across negative, neutral, and positive word stimuli, but the differences were quite small. Interaction effects were detected for each of the conditions as well: word valence x go/no-go condition x language, F(2, 9979) = 67.03, p < .001, η^2^ = .008; word valence x language, F(2, 9979) = 62.57, p < .001, η^2^ = .007; go/no-go x language, F(2, 9979) = 7.89, p = .005, η^2^ = .0004, go/no-go x word valence, F(2, 9979) = 26.21, p < .001, η^2^ = .003. Post hoc tests showed that for the no-go condition, accuracy scores were significantly higher for Japanese negative words compared to English negative words (Japanese Mean = 98.60; English Mean = 95.54; p < .001), a non-significant difference was observed for neutral words (Japanese Mean = 96.79; English Mean = 97.04), whereas accuracy scores were significantly lower for Japanese positive words compared to English positive words (Japanese Mean = 94.32; English Mean = 95.34; p < .001). For the go condition, all accuracy scores were non-significant between languages: negative (Japanese Mean = 99.85; English Mean = 99.55), neutral (Japanese Mean = 99.12; English Mean = 99.69), and positive (Japanese Mean = 99.75; English Mean = 99.15; see Fig 4).

**Fig 5:**
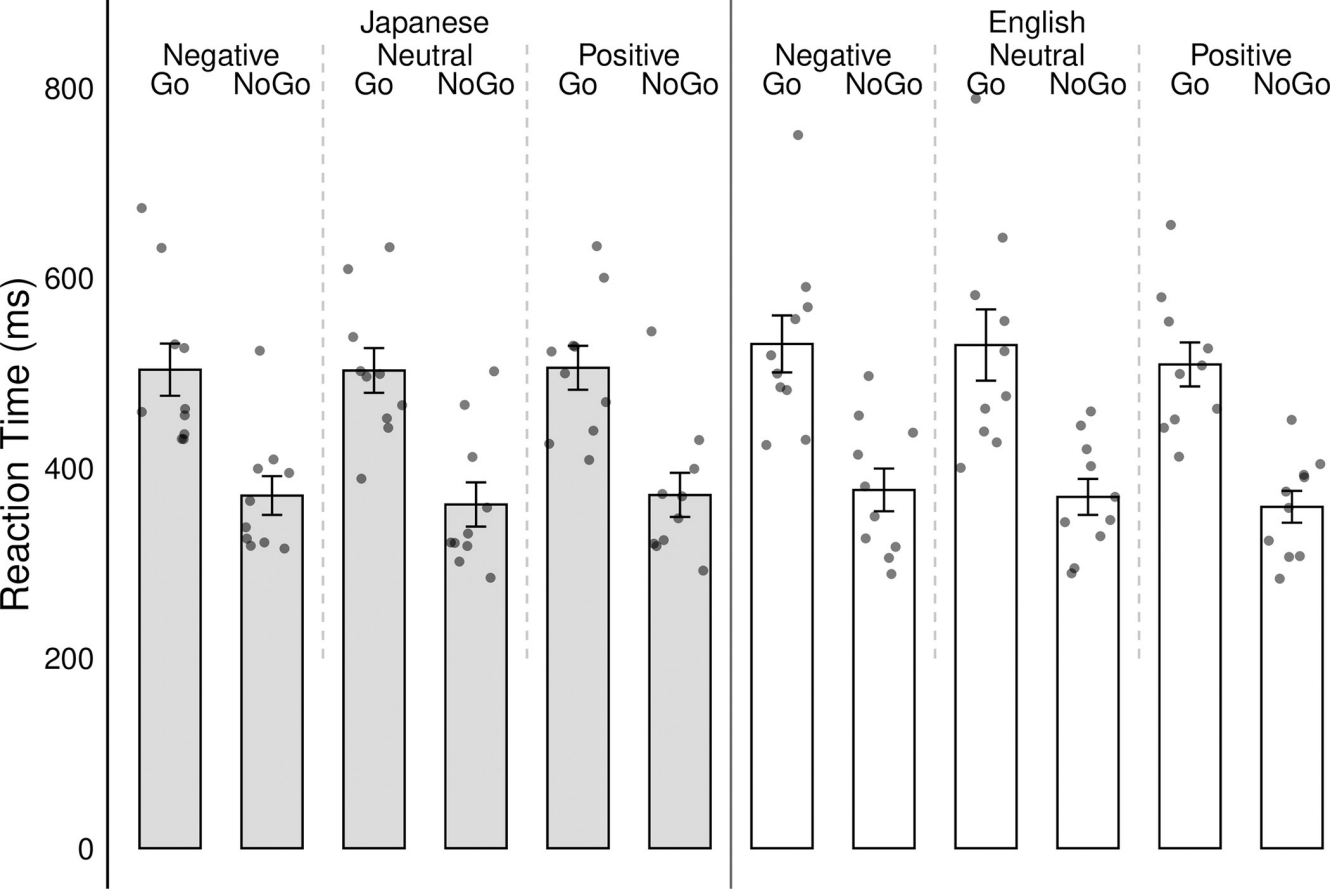
Reaction time (milliseconds) of each participant for language (English/Japanese), word valence (negative/neutral/positive) and go/no-go condition. Error bars denote standard errors of the mean. Each dot represents one participant.

Results on reaction times also showed statistically significant differences (see Fig 5).

The overall model was significant F(20, 9979) = 1829.56, p > .001 and explained roughly 79% of the variance (R^2^ = .785). Regarding language condition, a significant main effect was observed, F(1, 9999) = 5.79, p = .0161, η^2^ = .0004, indicating that RTs differed between English and Japanese instruction sets. Similarly, the go/no-go condition yielded a significant main effect, F(1, 9999) = 19352.80, p < .001, η^2^ = .4396, indicating substantial variation in RTs between conditions where participants were required to respond or withhold response. Additionally, for word valence, a significant main effect was found, F(2, 9988) = 20.84, p < .001, η^2^ = .0028, revealing differences in RTs across negative, neutral, and positive word stimuli. Interaction effects were detected for each of the conditions as well, with minute effects similar to those found in the accuracy scores: word valence x go/no-go condition x language, F(2, 9979) = 18.22, p < .001, η^2^ = .0007; word valence x language, F(2, 9979) = 68.22, p < .001, η^2^ = .003; go/no-go x language, F(2, 9979) = 388.62, p > .001, η^2^ = .008, go/no-go x word valence, F(2, 9979) = 7.70, p < .001, η^2^ = .0003. Post hoc tests found significantly faster reaction times for Japanese compared to English words for all emotion conditions in the no-go condition: negative (Japanese Mean = 387.69; English Mean = 364.72; p < .001), neutral (Japanese Mean = 363.34; English Mean = 350.72; p < .001), and positive (Japanese Mean = 374.25; English Mean = 363.43; p < .001). Whereas significantly slower reaction times for Japanese compared to English words were observed for all emotion conditions in the go condition: negative (Japanese Mean = 513.69; English Mean = 534.59; p < .001), neutral (Japanese Mean = 508.01; English Mean = 545.19; p < .001), and positive (Japanese Mean = 496.52; English Mean = 514.34; p < .001; see Fig 5).

## Discussion

The current study explored the extent to which the extended amygdala and amygdala mediate emotion and inhibitory control to emotionally valenced linguistic stimuli in a first or second language. Our results suggest, for the first time, that the left amygdala, right amygdala, left extended amygdala and right extended amygdala play an important role in mediating emotional linguistic stimuli in participant’s native language or in a second language when they are attempting to engage in inhibitory control, supported by a significant interaction for valence, go/no-go condition, and language for activity of all four ROIs. Furthermore, significant main effects for language and valence were observed for all four ROIs; however, a significant main effect for the go/no-go condition was only observed for the left and right extended amygdale and the right amygdala but not for the left amygdala.

Further examination of our findings using post hoc tests found that under conditions of inhibitory control participants displayed less activation in the extended amygdala and amygdala when processing emotional information in a second language compared to their native language. Specifically, we found increased activation in all four ROIs for Japanese words compared to English words when participants were engaging in inhibitory control (i.e., in the no-go condition) in the negative and positive emotion condition. However, a different pattern of results was observed in the go condition (i.e., when participants were not attempting to engage in inhibitory control) with increased activation in all four ROIs for English words compared to Japanese words in the negative emotion condition but decreased activity in each ROI for English words compared to Japanese words in the positive emotion condition (see Fig 3).

The current findings support previous findings regarding the role of the amygdala and extended amygdala in mediating emotion. Firstly, regarding the amygdala, past findings suggest that differences in right amygdala activation are observed when participants are presented with negative emotional stimuli [12] and that the left amygdala is active when exposed to emotionally positive [8, 10] or negative stimuli [12]. In the current study we found significant differences in activity of the right or left amygdala for emotional valence (positive, negative, or neutral linguistic stimuli). Secondly, regarding the extended amygdala, our findings also supported past findings in which pleasant (i.e., positive) or unpleasant (i.e., negative) stimuli produced differences in extended amygdala activation [7]. In the current study, we observed a significant main effect of valence (negative, or positive words) for both left and right extended amygdala activation.

Finally, the current results found significant effects for our behavioral measures, that is accuracy scores and reaction time for language, word valence and go/no-go conditions as supported by the significant interaction for valence, language, and go/no-go conditions for both behavioral measures. Furthermore, conditions of inhibitory control (no-go) versus non-inhibitory control (go) also appear to be important in the observed behavioral effects when separately examining language and emotional valence as significant interactions were observed for language (i.e., language x go/no go) and emotional valence (i.e., valence x go/no go) for both accuracy and reaction times.

Post hoc analyses of our behavioral measures explored whether participants would be better able to engage in emotional self-regulation (i.e., perform better in an inhibitory control task) in English compared to Japanese. We found that in the no-go condition participants displayed higher accuracy scores in Japanese compared to English for negative emotional stimuli but lower accuracy scores in Japanese compared to English for positive emotional stimuli (see Fig 4). These results suggest that participants may be better able to engage in inhibitory control when processing negative emotional information in their native language compared to a second language but less able to engage in inhibitory control in their native language when processing positive emotional information.

Notwithstanding the current findings, the limitations of the current study should be addressed. Future studies should increase the sample size used in the current study. Even though our sample size is consistent with studies using fMRI go/no-go tasks [29] and most published studies using fMRI where the median sample size is twelve participants [32] a larger sample size may allow for more robust conclusions. Although we attempted to address the smaller sample size in the current study using bootstrapping techniques, and while bootstrapping allows for a more reliable representation, it is still extracted from values of a small sample size.

## Conclusions

In conclusion, the current study suggests that the amygdala and extended amygdala activation may be affected by emotional-linguistic stimuli depending on which language the stimuli are presented under conditions of inhibitory control. Furthermore, our findings suggest that overt behavior (inhibitory control) was also affected by the emotional valence of our stimuli and the language of presentation through reaction time and accuracy scores in our go/no-go task. In conclusion, these results suggest that the amygdala and extended amygdala may play an important role in mediating emotion and inhibitory control when processing information in a native versus a second language.

## Supporting information

S1 Fig EPI signals for the right extended amygdala (panel a.), left extended amygdala (panel b.), right amygdala (panel c.), and left amygdala (panel d.). (TIF)
