# Supplementary figures and images for: An fMRI study examining the role of the extended amygdala and amygdala in emotion and inhibitory control in native versus second language processing
Source: PLoS One. 2024 Nov 4;19(11):e0310129. doi: 10.1371/journal.pone.0310129 (PMC11534241; doi:10.1371/journal.pone.0310129)

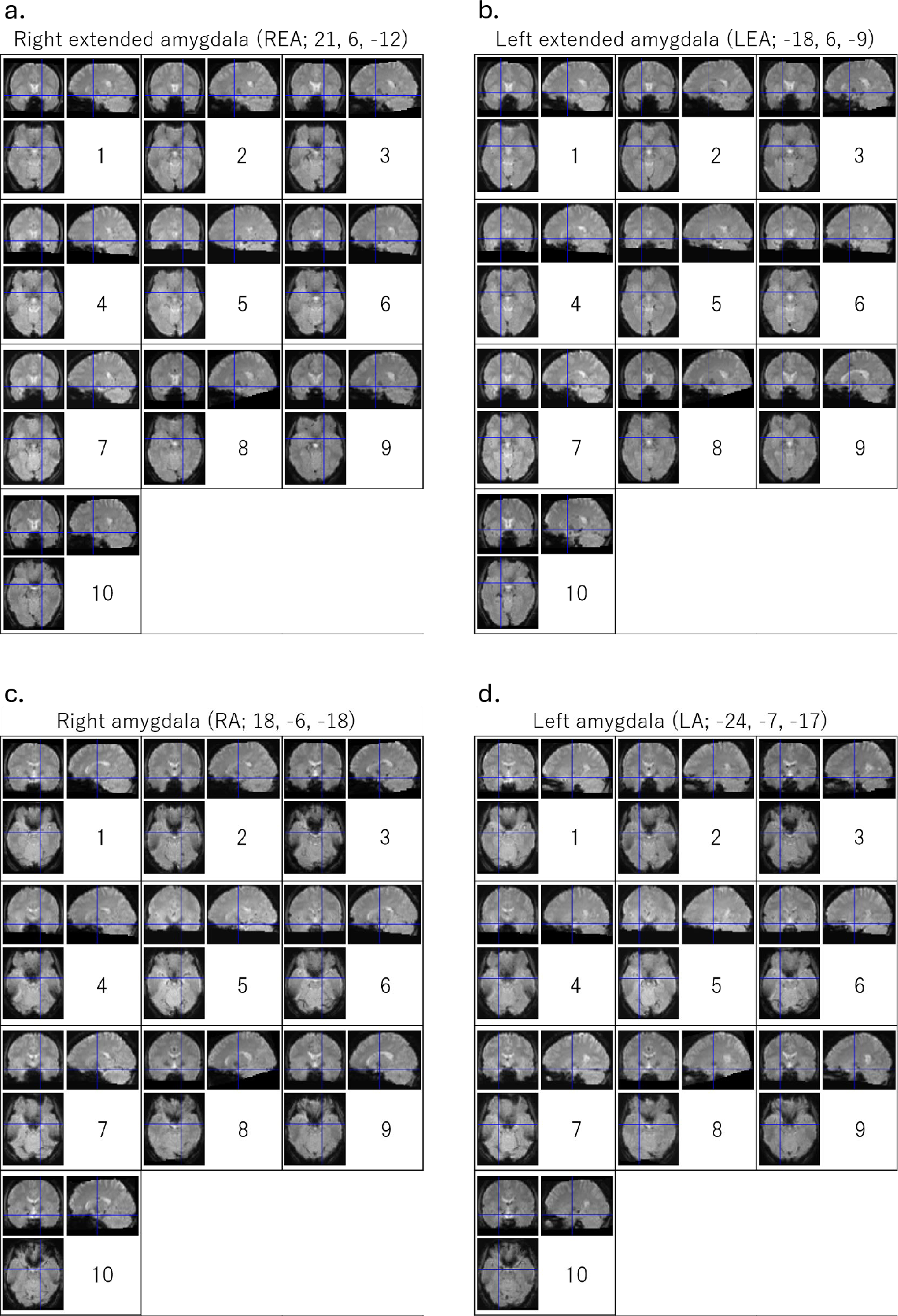

Supplement: S1 Fig — (TIF) [file pone.0310129.s001.tif]
